# Phase Variation of PorA, a Major Outer Membrane Protein, Mediates Escape of Bactericidal Antibodies by Neisseria meningitidis

**DOI:** 10.1128/IAI.01358-12

**Published:** 2013-04

**Authors:** Isfahan Tauseef, Youssif M. Ali, Christopher D. Bayliss

**Affiliations:** a Department of Genetics; b Department of Infection, Immunity, and Inflammation, University of Leicester, Leicester, United Kingdom; c Microbiology Department, Faculty of Pharmacy, Mansoura University, Mansoura, Egypt

## Abstract

Several outer membrane proteins of Neisseria meningitidis are subject to phase variation due to alterations in simple sequence repeat tracts. The PorA protein is a major outer membrane protein and a target for protective host immune responses. Phase variation of PorA is mediated by a poly-G repeat tract present within the promoter, leading to alterations in protein expression levels. N. meningitidis strain 8047 was subjected to serial passage in the presence of P1.2, a PorA-specific bactericidal monoclonal antibody. Rapid development of resistance to bactericidal activity was associated with a switch in the PorA repeat tract from 11G to 10G. Phase variants with a 10G repeat tract exhibited a 2-fold reduction in surface expression of PorA protein. A *mutS* mutant of strain 8047, with an elevated phase variation rate, exhibited a higher rate of escape and an association of escape with 10G and 9G variants, the latter having a 13-fold reduction in surface expression of PorA. We conclude that graduated reductions in the surface expression of outer membrane proteins mediated by phase variation enable meningococci to escape killing *in vitro* by bactericidal antibodies. These findings indicate how phase variation could have a major impact on immune escape and host persistence of meningococci.

## INTRODUCTION

Awide variety of surface structures and outer membrane proteins of a diverse range of bacteria species are subject to phase variation (PV) (1–4). Reversible and high-frequency alterations in expression of these surface molecules or epitopes may be mediated by mechanisms involving mutation, recombination, or differential methylation of promoter sequences (5–7). Phase variation may result in ON/OFF changes in expression or more graduated alterations. While selection for the ON phenotype usually involves a gain of function—adhesion, iron acquisition, complement resistance—the selective advantage associated with an OFF phenotype is more difficult to discern and demonstrate. One view is that antigen-specific antibody (Ab) responses are a major selective force acting on phase-variable antigens of bacterial pathogens and commensals (8).

Neisseria meningitidis is an obligate commensal of the upper respiratory tract of humans. Asymptomatic carriage occurs in 10% to 15% of the population, with carriage levels rising to 50% or more in certain groups such as university students and army recruits (9, 10). Meningococci can invade host tissues and cause clinically important infections such as septicemia and meningitis. Levels of disease in areas of endemicity are low—occurring at a rate of 1 to 5 cases per 100,000—but epidemics are observed in Africa with much higher rates of infection. Asymptomatic carriage of meningococci can last for 6 to 9 months and is associated with the induction of protective strain-specific immune responses (11–13). One of the major targets of these immune responses is the PorA protein, one of the most highly expressed outer membrane proteins of meningococci. PorA is a transmembrane protein with seven outer membrane loops (14). Two of these loops (VR1 and VR2) exhibit high levels of antigenic variation and are utilized for strain typing. The PorA protein is also a key vaccine candidate, despite generating only strain-specific protection, and a number of meningococcal vaccines contain this antigen, including one, Bexsero, which is nearing licensing (15, 16).

Multiple genes of N. meningitidis are subject to PV mediated by alterations in repeat tracts present within the reading frame or promoter. The rates of PV of genes containing mononucleotide repeats, but not tetra- or pentanucleotides, are increased 100- to 1,000-fold by mutations in mismatch repair genes (17, 18). Immune escape due to PV has been demonstrated for *lgtG* (19). This gene encodes a glucosyltransferase and modifies the structure of lipopolysaccharide (LPS), with addition of this moiety to the LPS generating resistance to the bactericidal activity of monoclonal antibody (MAb) B5. A poly-G repeat tract is present within the reading frame of *lgtG*, and OFF-to-ON switching caused by changes in this tract mediated escape of MAb B5 during multiple passages in a modified serum bactericidal assay. Escape was enhanced by a *mutS* mutation due to an increase in the rate of PV.

Many of the phase-variable genes of N. meningitidis encode outer membrane proteins. The *porA* gene contains a poly-G repeat between the −10 and −35 components of the promoter. Changes in the length of this repeat tract mediate alterations in the levels of expression of the PorA protein. While strains exhibit variation in the length of this repeat tract and phase variants have been isolated from meningococcal carriers (20), it is unclear whether these variants provide a selective advantage to meningococci. In this report, we demonstrate that escape of killing by a bactericidal monoclonal antibody targeted to the PorA protein, MAb P1.2 (21), is mediated by alterations in the repeat tract and that these alterations are associated with graduated changes in the level of surface expression of this protein.

## MATERIALS AND METHODS

### Bacterial strains and growth conditions.

N. meningitidis strain 8047 and a *mutS* mutant of this strain (8047 Δ*mutS*; [19]) were grown either on plates with brain heart infusion (BHI) agar supplemented with Levinthal's medium (10% vol/vol) or on chocolate agar plates (Oxoid) in 5% CO_2_ at 37°C. Meningococcal cells were prepared for examining protein expression levels following growth either overnight or to the mid-log phase in 10 ml of BHI broth at 37°C on an orbital shaker.

### Modified serum bactericidal assay.

A continuous modified bactericidal assay was described previously and was utilized for all the assays described here (19). Briefly, meningococci grown overnight on agar plates were resuspended in phosphate-buffered saline supplemented with 0.5 mM MgCl_2_ and 0.9 mM CaCl_2_ (pH 7.4) (PBSB) and utilized for preparation of a 500-μl inoculum in PBSB–0.1% glucose. This inoculum was mixed with 500 μl of PBSB–0.1% glucose containing 10% human serum (an adult pooled serum from healthy donors) and various dilutions of MAb P1.2 (Poolman) (MN16C13F4; obtained from the National Institute for Biological Standards and Control [NIBSC] and resuspended to generate a stock with a concentration of 1:5,000 for use in a whole-cell enzyme-linked immunosorbent assay [ELISA] [21]). Samples were incubated for 2 h at 37°C in 5% CO_2_ (passage 1 [P1]). An aliquot of 500 μl was then removed and mixed with 500 μl of a freshly prepared mix of human serum and MAb in PBSB–0.1% glucose (P2). Subsequent passages were performed in a similar manner. Serial dilutions of samples were performed after each passage and spread onto BHI-Levinthal's agar plates for assessing bacterial numbers.

### Genotypic and phenotypic analysis of PorA variants.

Output populations from the modified serum bactericidal assay were initially assessed by colony immunoblotting using a 1:2,000 dilution of MAb P1.2 as a primary antibody and a previously described methodology. Boiled lysates were prepared from phase variants, detected by colony immunoblotting, or from input colonies either directly or following regrowth on BHI/Levinthal's agar plates. Where immunoblotting failed to detect phase variants, colonies were selected at random from input and output plates and utilized for preparation of boiled lysates. Amplification of the PorA repeat tract was then performed using primers porA-p21-rev (5′GGCCGGCTTTGATTTCGCCGTACAG) and either porA-p72 (5′GCACGAGGTCTGCGCTTGAATTG) or porA-p72–6-carboxyfluorescein (FAM) within a standard PCR and the following cycling conditions: 94°C for 1 min, 50°C for 1 min, and 72°C for 1 min, for 25 cycles. PCR products were analyzed either by sequencing using primer porA-p72 and BigDye (PerkinElmer Life Sciences) or by GeneScan.

### Analysis of PorA expression levels.

Protein expression levels were assessed in variants grown overnight in BHI broth supplemented with Levinthal's medium. Following growth, cells were harvested by centrifugation and the cell pellet was washed by resuspension in PBS. For Western blot analyses, the cell concentration was adjusted to 1 × 10^9^ cells/ml and then a known amount of cells was pelleted and resuspended in 1× SDS-PAGE loading buffer. Samples were subjected to electrophoresis on 10% PAGE gels followed by transfer to nitrocellulose membranes and probing with MAb. Duplicate gels were stained with Coomassie blue to check for equal loading of proteins.

For the whole-cell ELISA, the cell pellet was resuspended in 0.05% formalin–PBS and incubated for 1 h. After fixation, the cell pellet was washed twice with PBS and resuspended in coating buffer (15 mM Na_2_CO_3_, 35 mM NaHCO_3_, pH 9.6) and the concentration was adjusted to an optical density at 550 nm (OD_550_) of ∼0.5. An aliquot of 100 μl of fixed cells was added to each well and incubated overnight at 4°C. Blocking was performed by addition of 1% bovine serum albumin (BSA)–Tris-buffered saline (TBS) (140 mM NaCl, 10 mM Tris, pH 7.4) plus 1.5 mM sodium azide for 1 h followed by three washes with washing buffer (TBS with 0.05% Tween 20). Dilutions of MAb P1.2 in 100 μl of PBST-BSA (PBS containing 0.1% Tween 20 and 2% bovine serum albumin) were added to appropriate wells and incubated for 1 h at room temperature. Similar washing and incubation steps were performed with a 1:3,000 dilution of anti-mouse alkaline phosphatase. Bound antibody was detected by addition of a substrate solution containing 5-bromo-4-chloro-3-indoylphophate-ntroblue tetrazolium (PerkinElmer Life Sciences) and measurement of the OD_405_ in a spectrophotometer.

For flow cytometry, cells were harvested from 200 μl of an overnight culture, washed twice with TBS plus 0.05% Tween 20, and resuspended in 150 μl of a 1:50 dilution of MAb P1.2 in PBST-BSA. Samples were incubated for 1 h on ice and washed twice with washing buffer prior to addition of 150 μl of a 1:100 dilution of an anti-mouse IgG-fluorescein isothiocyanate (FITC) conjugate and incubation for 1 h on ice. Bacterial cells and bound antibody were washed twice and then resuspended in 750 μl of PBST-BSA plus 0.05% formalin. The negative control consisted of cells incubated with the secondary antibody alone. The samples were analyzed on a fluorescence-activated cell sorter (FACS) scanner, and a mean fluorescent intensity (MFI) was determined for each sample.

## RESULTS

### Phase variation of *porA* mediates escape of N. meningitidis strain 8047 from the bactericidal activity of a PorA-specific monoclonal antibody.

A modified serum bactericidal assay was developed for detecting immune escape by meningococci (19). This assay involves subjecting a relatively large inoculum (5 × 10^3^ to 5 × 10^6^ CFU) of meningococcal cells to multiple sequential passages in the presence of a bactericidal antibody and human serum as a source of active complement. Surviving cells, termed escape variants, are tested for alterations in relevant genes. This assay was utilized in a demonstration that PV due to a repeat tract present within the reading frame of *lgtG* mediated escape of MAb B5, a bactericidal antibody specific for an LPS epitope (19). The *porA* gene is also subject to PV due to a poly-G repeat tract present within the promoter resulting in alterations in the levels of protein expression rather than a simple ON/OFF switch (20). PorA-negative variants with changes in a 7A tract within the 5′ end of the reading frame have also been observed (20). In order to investigate whether PV of *porA* could mediate immune evasion, N. meningitidis strain 8047 was subjected to selection with MAb P1.2, a bactericidal antibody specific for VR2 of the PorA protein—with type P1.5-2,2-2—of this strain. This strain contains an 11G repeat tract located between the −35 and −10 elements of the *porA* promoter. Initial experiments detected escape variants of various types, including 9G and 10G phase variants, a change in the 7A tract to 8A, and alterations in VR2 (data not shown). These experiments indicated that our stock of this strain contained a range of genetic variants; hence, a new stock was generated from a single colony and utilized in the assays described here. All variation was restricted to the 11G tract in subsequent experiments, indicating that variation due to changes in the 7A tract was negligible in these assays, possibly due to a significantly lower mutation rate being associated with the latter tract (data not shown).

**Fig 1:**
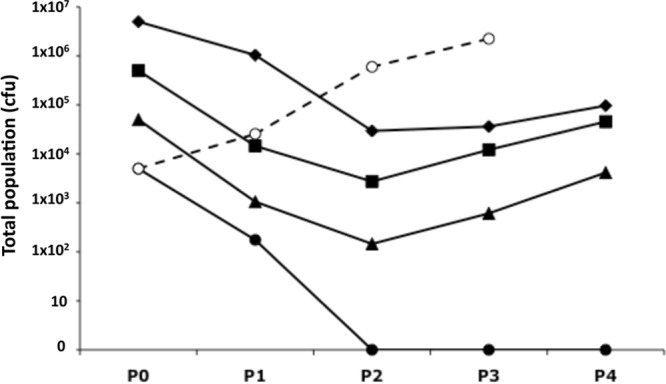
Escape of Neisseria meningitidis strain 8047 from the PorA MAb P1.2-mediated serum bactericidal activity. Strain 8047 was incubated in the presence of 5% human serum diluted in 1 ml of PBSB containing 0.1% glucose and in 10 μl of a 1:4 dilution of MAb P1.2. Inocula for the first passage were prepared from an overnight culture grown on BHI plates. Subsequent passages (after the first passage) were performed by mixing 500 μl of the passaged population with an equal volume of PBSB containing human serum and antibody. Each passage was 2 h. The *x* axis represents the number of passages performed, with P0 being the inoculum. Filled diamonds, inoculum of 5 × 10^6^ CFU with MAb; filled squares, inoculum of 5 × 10^5^ CFU with MAb; filled triangles, inoculum of 5 × 10^4^ CFU with MAb; filled circles, inoculum of 5 × 10^3^ CFU with MAb; open circles and dashed line, inoculum of 5 × 10^3^ CFU without MAb.

Inoculum populations of three different sizes were subjected to multiple cycles of incubation in the presence of 5% human serum as a source of complement and 10 μl of a 1:4 dilution of the stock solution of MAb P1.2 obtained from NIBSC (Fig. 1). A reduction of up to 47-fold relative to the inoculum was detected in the populations incubated with antibody, indicative of killing by the MAb. In the absence of antibody, no reduction was observed, demonstrating that the human serum did not contain any bactericidal activity for this meningococcal strain (Fig. 1). The lowest inoculum of 5,000 CFU was completely eliminated by passage. For the other inoculums, no further reductions were detected after passage 2, indicating that these bacterial populations had developed resistance to the MAb and were beginning to replicate at rates similar to those seen with the control population.

Preliminary experiments demonstrated that immunoblotting could readily detect lightly stained PorA-negative OFF variants and that these variants had either a 9G repeat tract or changes in the 7A tract (data not shown). Immunoblot analyses were, therefore, performed on the inoculum and output populations. All of the populations exhibited similar levels of high reactivity, but no phase variants with negative or reduced staining were reliably observed (data not shown). These experiments indicated that escape was not due to generation of OFF variants of the *porA* gene but could not distinguish whether there had been a reduction in protein expression due to PV. In order to determine whether PV had occurred, a number of randomly isolated colonies were analyzed for alterations in the *porA* repeat tract (experiment 1, Table 1). All the colonies from the inoculum population or the population incubated without antibody had an 11G repeat tract, associated with high expression. Populations subjected to MAb selection exhibited a switch from a mixture of 11G and 10G variants in P1 to populations of 10G variants in subsequent passages. While only a small number of variants have been analyzed, these experiments were consistent with escape being due to survival and proliferation of 10G *porA* variants.

**Table 1:** Repeat tract lengths of *porA* for single colonies following selection with MAb P1.2*^a^*

Inoculum size/selection	Tract length	No. of colonies							
		Expt 1		Expt 2			Expt 3		
		Inoculum (P0)*^b^*	P1/P4	Inoculum (P0)*^b^*	P1	P4	Inoculum (P0)*^b^*	P1	P3/P4
5 × 10^6^/+MAb	11G	5	5	ND	ND	ND	4	0	0
	10G	0	4	ND	ND	ND	0	8	8
5 × 10^5^/+MAb	11G	5	5	4	1	0	4	1	0
	10G	0	5	0	11	18	0	3	8
5 × 10^4^/+MAb	11G	5	1	ND	ND	ND	4	—	0
	10G	0	7	ND	ND	ND	0	—	4
5 × 10^3^/+MAb	11G	5	3	4	0	0	ND	ND	ND
	10G	0	0	0	—	—	ND	ND	ND
No MAb*^c^*	11G	5	5	4	6	8	4	8	4
	10G	0	0	0	0	0	0	0	0

a Experiments 1 and 2 utilized a 1:4 dilution of MAb, while experiment 3 utilized nondiluted MAb. —, no samples were collected. ND, no data.

b Note that the same inoculum was used for each of the different inoculum sizes.

c An inoculum size of 5 × 10^3^ was used for experiments 1 and 2 and 5 × 10^4^ for experiment 3.

### Population size and antibody concentration have a major impact on immune escape.

**Fig 2:**
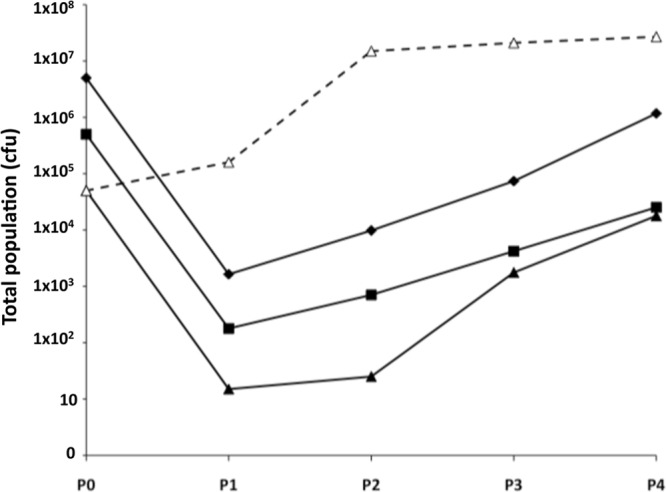
Influence of a high antibody concentration on escape of PorA MAb P1.2 by N. meningitidis strain 8047. Passage experiments were performed as described for Fig. 1 except that each passage contained 10 μl of nondiluted MAb P1.2. Filled diamonds, inoculum of 5 × 10^6^ CFU with MAb; filled squares, inoculum of 5 × 10^5^ CFU with MAb; filled triangles, inoculum of 5 × 10^4^ CFU with MAb; open triangles and dashed line, inoculum of 5 × 10^4^ CFU without MAb.

The phase variation rate influences the numbers of phase variants in a particular population and, hence, the size of the population mediating immune escape. As escape was observed with a population of 5 × 10^4^ CFU but not 5,000 CFU (Fig. 1), a second experiment was performed with an intermediate inoculum (5 × 10^5^ CFU) and a low inoculum (5 × 10^3^ CFU). Escape was again observed with the intermediate but not the low inoculum (see Fig. S1 in the supplemental material) and was due to the accumulation of 10G variants (experiment 2, Table 1). A lower proportion of 11G variants was observed in this assay than in experiment 1 (Table 1), probably due to the higher level of killing associated with this assay (compare Fig. 1 and Fig. S1 in the supplemental material) and, hence, to more-rapid turnover from 11G to 10G variants. A low inoculum incubated without antibody exhibited continuous growth, indicating that elimination of the low inoculum population was due to killing by MAb P1.2 and the absence of phase variants rather than to an effect of the complement source. As the antibody may have been limiting killing in these assays, another experiment was performed with 10 μl of the undiluted stock antibody and three different inoculum sizes (Fig. 2). Populations were reduced to a minimal level after one passage and then in subsequent passages exhibited continuous growth.

Colonies were examined for alterations in the *porA* repeat tract. Only 11G variants were detected in the inoculum and populations incubated without antibody, while a switch from a mix of 11G and 10G in P1 to only 10G was detected in the MAb-selected populations (experiment 3, Table 1). These experiments provided further evidence of the role of 10G variants in mediating escape of N. meningitidis strain 8047 from MAb P1.2-mediated killing.

### A higher phase variation rate increases escape of MAb P1.2.

**Fig 3:**
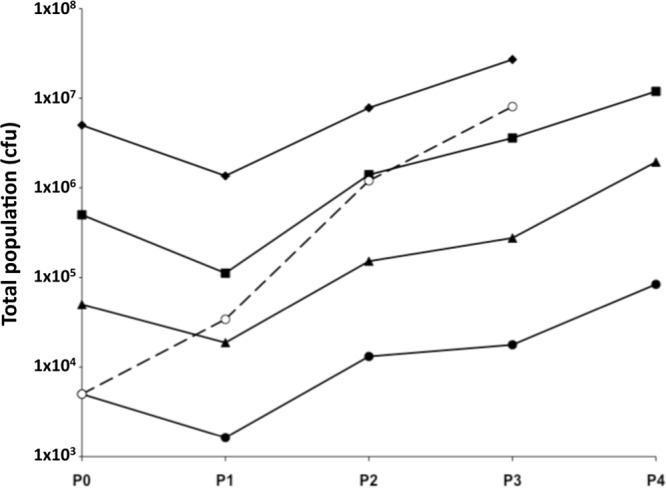
A higher phase variation rate increases escape of PorA MAb P1.2. Passage experiments were performed as described for Fig. 1 except that this experiment utilized a mutator strain (8047 Δ*mutS*) with a 1,000-fold-higher phase variation rate than the parent, strain 8047. Filled diamonds, inoculum of 5 × 10^6^ CFU with MAb; filled squares, inoculum of 5 × 10^5^ CFU with MAb; filled triangles, inoculum of 5 × 10^4^ CFU with MAb; filled circles, inoculum of 5 × 10^3^ CFU with MAb; open circles and dashed line, inoculum of 5 × 10^3^ CFU without MAb.

The low PV rate of strain 8047 could have resulted in the absence of escape variants with a 9G *porA* repeat tract. In order to investigate the influence of PV rate and to increase the potential for production of 9G phase variants, we examined escape of the P1.2 MAb by a *mutS* mutant of strain 8047, which has a 1,000-fold-higher PV rate than the wild-type strain (19). Using 10 μl of a 1:4 dilution of the MAb, resistance was observed to develop rapidly and only a limited amount of killing was observed in P1 (Fig. 3). Even a low inoculum population of 5 × 10^3^ CFU exhibited survival, indicating the presence of a high level of MAb-resistant variants. Colony immunoblot analyses were performed on the inoculum and output populations. High levels of nonreactive OFF variants were detected in the populations incubated with antibody but not in those without antibody (data not shown). The proportion of nonreactive variants increased from ∼10% in P1 to 50% to 70% by P4 in populations incubated with antibody (data not shown). Analysis of the repeat tracts of 19 negative variants indicated the presence of a 9G repeat tract (Table 2). Positive variants from the inoculum population or the passage 4 (P4) population incubated without antibody (*n* = 8) all had an 11G tract, while those from P1 (*n* = 6) or P4 (*n* = 9) of MAb P1.2-selected populations had a 10G tract (Table 2). These results indicated that the *mutS* mutant exhibited a higher rate of escape due to the presence of high levels of 10G and 9G variants in the inoculum population. Furthermore, there was a suggestion of 9G variants outcompeting 10G variants during prolonged selection with this MAb.

**Table 2:** Repeat tract lengths of *porA* variants of a meningococcal mutator strain following selection with MAb P1.2*^c^*

Inoculum size/selection	Tract length*^a^*	No. of colonies		
		Inoculum (P0)*^b^*	P1	P4
5 × 10^6^/+MAb	11G	5	0	—*^c^*
	10G	0	4	—
	9G	0	2	—
5 × 10^5^/+MAb	11G	5	0	—
	10G	0	1	—
	9G	0	5	—
5 × 10^4^/+MAb	11G	5	0	0
	10G	0	7	1
	9G	0	0	12
5 × 10^3^/+MAb	11G	5	0	—
	10G	0	1	—
	9G	0	0	—
5 × 103/no MAb	11G	5	3	—
	10G	0	0	—
	9G	0	0	—

a 10G and 11G were positive on colony immunoblots, and 9G variants were negative.

b Note that the same inoculum was used for each of the different inoculum sizes.

c —, no samples were collected.

### Escape variants exhibit an intermediate or low level of PorA expression.

This series of experiments indicated that escape could be mediated by 10G variants, which exhibited a high level of reactivity with MAb P1.2 in immunoblot analyses, or by nonreactive 9G variants. As it was unclear if escape by 10G variants was due to differential expression of the *porA* gene, the levels of PorA protein expression were examined in a parental 11G variant of N. meningitidis strain 8047 and 10G and 9G variants obtained during selection experiments.

**Fig 4:**
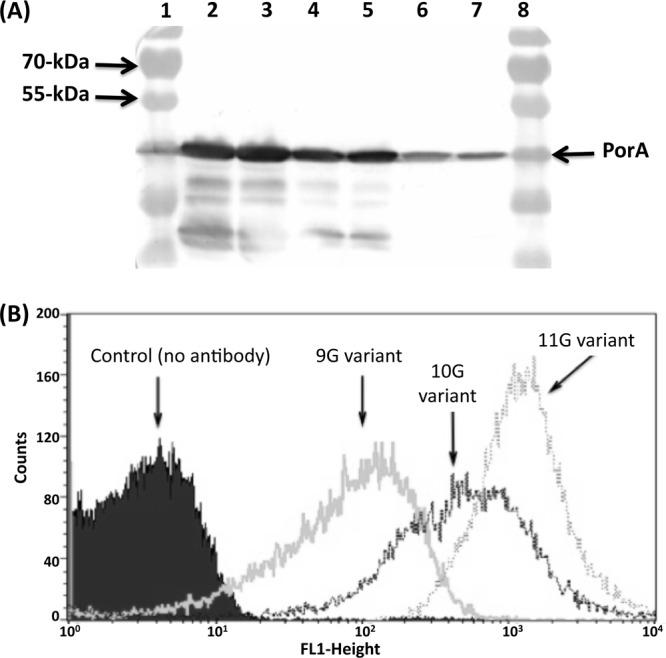
Phase variants of *porA* differ in the levels of total and surface expression of PorA protein. Meningococcal cultures were prepared from different *porA* phase variants of strain 8047. (A) Total expression of PorA protein was determined using whole-cell lysates. Lysates were electrophoresed on a 10% SDS-PAGE gel and subjected to Western blotting followed by probing of membranes with a 1:2,000 dilution of MAb P1.2 and detection with an alkaline phosphatase-conjugated antibody. Lanes 1 and 8, size standards; lanes 2 and 3, one 11G phase variant loaded in duplicate; lanes 4 and 5, two different 10G phase variants; lanes 6 and 7, two different 9G phase variants. (B) Surface expression of PorA was evaluated using whole cells. Meningococcal cells were incubated with a 1:50 dilution of MAb P1.2 followed by incubation with a 1:100 dilution of an anti-mouse FITC conjugate antibody. Cells were then fixed and subjected to FACS analysis. Mean MFI values were determined for each phase variant following incubation with MAb and for an 11G variant without MAb. An overlay of the histograms for each sample is shown.

Western blots of whole-cell lysates were probed with MAb P1.2. A distinct difference between these variants in the levels of expression was detected (Fig. 4A). Quantification of the Western blot analysis results indicated 1.2- and 3-fold-lower levels of expression in 10G and 9G variants, respectively, than in an 11G variant. A whole-cell ELISA of formalin-fixed meningococcal cells was utilized to examine surface expression, and similar differential levels of expression between these variants were detected (see Fig. S2 in the supplemental material), with 10G and 9G variants having a 2- or 6-fold-lower level of expression than 11G variants. This differential surface expression was confirmed by FACS analysis (see Fig. S3 in the supplemental material), and an overlay of the histograms demonstrated the lower levels of surface expression present in 10G and 9G variants (Fig. 4B). Comparison of the mean MFI values detected a 2.2-fold-lower level of expression in 10G variants than in 11G variants and a 13-fold difference between 9G and 11G variants. Overall, these experiments indicated that 10G variants had an intermediate level of surface expression of the PorA protein compared to 11G variants and that 9G variants had a low level of expression.

### Phase variants with an intermediate *porA* expression level have a fitness disadvantage relative to negative variants during immune escape.

**Fig 5:**
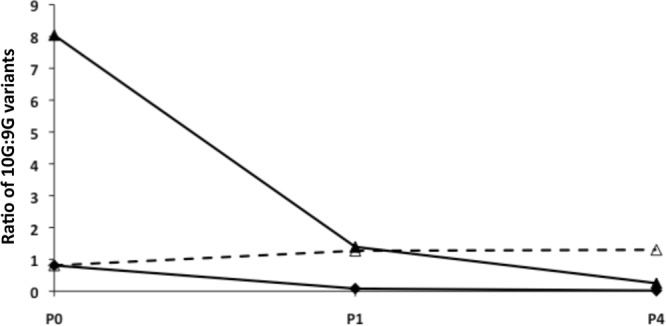
Phase variants with low levels of PorA expression outcompete higher levels of expression during escape of MAb P1.2. Variants with medium (10G) and low (9G) expression levels were grown overnight and used for inoculum preparation. Two different inoculums containing different ratios of the two variants were tested in an escape assay containing 10 μl of concentrated MAb P1.2 (see Fig. 1 for incubation conditions). Appropriate dilutions of passaged populations were grown overnight on BHI agar plates and subjected to immunoblotting with MAb P1.2 to detect each of the phase variants (positive colonies were assumed to be 10G variants and negative colonies 9G variants). The ratio of 10G/9G variants for passages 1 and 4 were calculated from these immunoblot analyses. Filled triangles, inoculum with 9:1 ratio of 10G/9G variants plus MAb; filled diamonds, inoculum with 1:1 ratio of 10G/9G variants plus MAb; open triangles and dotted lines, inoculum with 1:1 ratio of 10G/9G variants without MAb.

The intermediate level of expression of the PorA protein in 10G variants suggested that these variants could bind significant levels of MAb P1.2 and may be susceptible to some level of killing by this antibody. Furthermore, the relative expression levels and results seen with the *mutS* mutant suggested that 9G variants would have a selective advantage compared to 10G variants. An initial assay was performed to determine whether replication of 10G variants was inhibited by MAb P1.2. A limited amount of killing and a reduction in the growth of 10G variants were observed during passage with either an intermediate level of MAb (see Fig. S4 in the supplemental material) or a high level (data not shown). The majority of surviving variants retained a 10G repeat tract (data not shown), although negative phase variants were detected in P4 at a frequency of 1:2,000 when an inoculum of 5 × 10^5^ CFU and concentrated antibody were utilized. A competition experiment was then performed in which the relative abilities of 9G and 10G variants to survive and replicate in the presence of MAb were examined (Fig. 5). The 10G variant was completely replaced by the 9G variant, whether at a ratio of 1:1 or 9:1 of 10G/9G. In the absence of antibody, the ratio of the two variants was static. These results indicated that MAb P1.2 has a bacteriostatic effect on 10G variants but not on 9G variants and hence that a 9G phenotype becomes dominant in the population.

## DISCUSSION

A wide variety of surface structures in a diverse range of bacterial commensals and pathogens are subject to alterations in expression due to PV. These PV events are thought to facilitate immune escape, but there is limited evidence to support this contention. Some PV events generate ON/OFF expression states for a particular structure, while others modulate the level of expression. The ON-to-OFF switching of a particular epitope on meningococcal LPS was previously shown to mediate escape of a specific bactericidal MAb (19). We demonstrate here that phase-variable alterations in the levels of surface expression of an outer membrane protein, PorA, can also mediate escape of a bactericidal MAb and we speculate on the importance of this phenomenon for host persistence of meningococci.

The role of PV in mediating escape of antibodies was examined using a modified serum bactericidal activity assay. This assay utilizes a large inoculum so that rare variants in a population are present and a serial incubation in order to prevent exhaustion of the bactericidal antibody or complement source. This assay mimics survival of a bacterial population on a mucosal surface and subject to continual and prolonged exposure to a host immune response. Escape of a bactericidal antibody specific for the PorA protein of N. meningitidis strain 8047 was readily observed in this assay. Escape was due to phase variants with a 10G repeat tract and a 2-fold reduction in surface expression of the PorA protein as detected by immunofluorescence. A minimal population size of 5 × 10^4^ CFU was required for escape to occur with this strain. This inoculum size was reduced to 15 CFU by the end of P1 with the highest antibody concentration (Fig. 2). The doubling time in these assays is ∼30 min, and each passage is 2 h; thus, the inoculum may have contained a single PorA-resistant variant. As only 10G phase variants were detected in P4, these data indicate that 10G phase variants were present within the inoculum at a frequency of ∼2 × 10^−5^. A similar PV frequency has been detected for other genes subjected to PV mediated by mononucleotide repeat tracts in meningococci (17, 18).

Immune escape due to a lower level of surface expression has been detected with other meningococcal antigens. The fHBP protein is a major vaccine target and is known to exhibit strain-to-strain variations in expression due in part to differing levels of a transcriptional activator (22, 23). Strains with low expression of fHBP exhibit resistance to bactericidal antibodies directed against this protein (24, 25). Similarly, a synergistic effect on meningococcal killing was obtained using combinations of antisera specific for antigens expressed at a low level whereas each antiserum alone exhibited minimal levels of bactericidal activity (26). These results suggest that a specific level of surface expression is required for complement-mediated killing of meningococci, a process influenced by the antibody classes and clustering of C1q molecules on the bacterial surface (24, 27). Our findings suggest that PV of the PorA protein, a major outer membrane protein, enables meningococci to escape killing by bactericidal antibodies due to a reduction in the concentration of surface-bound antibody and hence reduced complement activation.

Escape due to phase variants with a 9G repeat tract and a 13-fold reduction in surface expression of PorA protein (as detemined by FACS analysis) were readily detected with a *mutS* mutant but not a wild-type strain. A switch from 11G to 9G would require two switching events; hence, variants would be expected at a frequency of 2.5 × 10^−9^ in a wild-type population. The absence of 9G variants was therefore not unexpected, as the largest inoculum size (5 × 10^6^) would not contain variants. A *mutS* mutant can increase the PV rate of mononucleotide repeat tracts 1,000-fold (17–19); hence, the frequency of 10G variants would be 5 × 10^−2^ and that of 9G variants would be 2.5 × 10^−4^, meaning that 9G variants would be present even in an inoculum of 5,000 CFU. A more intriguing issue is why variants were not readily observed when an inoculum of 10G variants was utilized, given that 9G variants can outcompete 10G variants. Assuming a PV rate for a frequency of 10G to 9G switching of 5 × 10^−5^, 25 variants would be expected in the 5 ×10^5^ inoculum and none in the smaller population size. If the 9G variants had a doubling time of 1 h, then 800 variants would be present by the end of the assay and a frequency of 1:6,000 for a total population of 5 × 10^6^. This is in agreement with the observed frequency of 9G variants of 1:2,000. A critical aspect of this assay is that the 10G variants were much less susceptible to MAb P1.2-mediated bactericidal activity; hence, detection of the replacement of 10G by 9G variants may require more passages and a higher antibody concentration.

Carriage of meningococci is known to induce a strain-specific serum bactericidal immune response, which can protect against infection (13). A significant proportion of this response is directed against the PorA protein. There is some evidence of a mucosal response, but it is less clear if this response interferes with meningococcal growth (28, 29). Our results suggest that prolonged carriage of meningococci may be associated with PV for variants with a lower level of PorA expression. A weak immune response, as present on mucosal surfaces, may select for variants with small reductions—2-fold—in PorA expression, while stronger responses may select for larger reductions. As mucosal immune responses are likely to be weak, we would anticipate that strains with very low expression (i.e., 9G variants) would be rare whereas 10G variants may accumulate as a function of the length of persistence.

The role of the PorA protein in meningococcal disease is unclear. Meningococcal strains lacking a PorA protein have occasionally been isolated from disease cases, suggesting that the PorA protein is not essential for meningococcal disease (30). However, there have been few studies of the PV status of PorA in disease isolates or following vaccination with PorA-containing vaccines. One study by van der Ende et al. (20) observed PV of PorA at a lower expression level, between throat and invasive isolates, in only 1 of 11 patients. Isolates from at least four of these patients exhibited high-level expression of the PorA protein. Alcalá et al. (31) examined 47 non-PorA typeable clinical isolates and found two with alterations in an A tract in the reading frame and one with a 9G tract in the promoter. Devoy et al. (32) examined 2,358 group B isolates from the New Zealand epidemic caused by an ST41/44 strain with an P1.7-2,4 PorA protein; they found only 156 nonserotypeable isolates, of which only 4 had alterations in the repeat tract of the homlogous PorA protein (3 with 9G and 1 with 10G). One of these 9G phase variants was subsequently shown to be resistant to the bactericidal effects of sera from individuals vaccinated with a homologous PorA protein (33), indicating that a reduction in PorA expression could mediate immune evasion. Gorla et al. (34) examined 42 vaccine failures from Brazil and found three with lower levels of expression of a heterologous PorA protein due to the presence of a 10G tract. These studies show that variants with lower levels of PorA expression due to changes in the G tract, as observed here, can be detected in clinical isolates and following vaccination. However, as the levels of anti-PorA antibodies were not assessed, it is not clear whether these changes in expression are due to immune evasion as predicted by our *in vitro* results. Indeed, these studies suggest that PorA-phase-variation-mediated immune escape of disease-associated or vaccine-induced immune responses is rare. Immune escape during disease may be rare because disease is thought to occur most frequently soon after colonization and before an immune response can develop. It is also possible that the initial screening with antibodies may have failed to detect variants with small reductions in PorA expression; hence, phase variation is underreported. It is still unclear, therefore, whether PV of PorA at intermediate levels (i.e., 10G variants) may facilitate invasive disease or vaccine failure by enabling escape of host-induced immune responses. Hence, a more comprehensive determination of the expression status of the *porA* gene in invasive isolates and isolates from vaccinated population will be important for understanding if phase variation contributes to immune evasion.

In summary, we provide evidence that phase-variable changes in the levels of expression of an outer membrane protein of meningococci can facilitate escape of antigen-specific bactericidal antibody responses. Critically, we observe escape due to small as well as large reductions in surface expression of this protein.

## Supplementary Material

Supplemental material
