# Supplementary material for: Phase Variation of PorA, a Major Outer Membrane Protein, Mediates Escape of Bactericidal Antibodies by Neisseria meningitidis
Source: Infect Immun. 2013 Apr;81(4):1374–80. doi: 10.1128/IAI.01358-12 (PMC3639595; doi:10.1128/IAI.01358-12)
Supplement: Supplemental material [file supp_81_4_1374__index.html]

Supplemental material 

# Phase Variation of PorA, a Major Outer Membrane Protein, Mediates Escape of Bactericidal Antibodies by Neisseria meningitidis

## Supplemental material

**Files in this Data Supplement:**

- Supplemental file 1 -

  Fig. S1. Population size influences escape of PorA MAb P1.2 by *N. meningitidis* strain 8047. Fig. S2. Whole-cell ELISA representing the level of PorA surface expression in three *porA* variants of strain 8047. Fig. S3. Detection by FACs of binding of PorA MAb P1.2 to phase variants of *N. meningitidis* strain 8047. Fig. S4. PorA expression level influences escape of PorA MAb P1.2.

  PDF, 766K
