# Supplementary material for: Phase Variation of PorA, a Major Outer Membrane Protein, Mediates Escape of Bactericidal Antibodies by Neisseria meningitidis
Source: Infect Immun. 2013 Apr;81(4):1374–80. doi: 10.1128/IAI.01358-12 (PMC3639595; doi:10.1128/IAI.01358-12)
Supplement: Supplemental material [file IAI.01358-12_zii999090081so1.pdf]

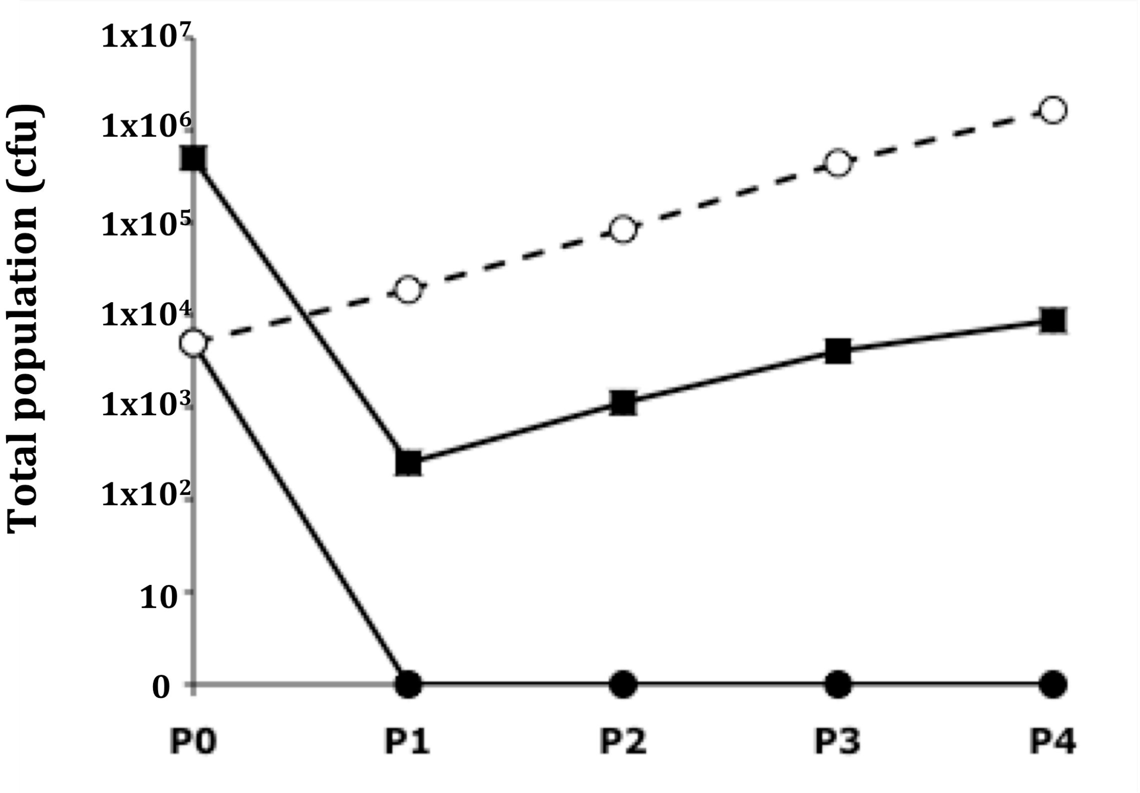

Supp. Figure 1. **Population size influences escape of PorA mAb P1.2 by *N. meningitidis* strain 8047.** Strain 8047 was incubated in the presence of 5 % human serum diluted in 1 ml of PBSB containing 0.1% glucose, and 10 µl of a 1:4 dilution of mAb P1.2. Inocula for the first passage were prepared from an overnight culture grown on BHI plates. Subsequent passages (after first passage) were performed by mixing 500 microlitres of the passaged population with an equal volume of PBSB containing human serum and antibody. Each passage was 2 hours. The X-axis represents the number of passage performed with P0 being the inoculum. Filled squares, inoculum of $5x10^5$ cfu with mAb; filled circles, inoculum of $5x10^3$ cfu, with mAb; open circles and dashed line, inoculum of $5x10^3$ cfu, no mAb.

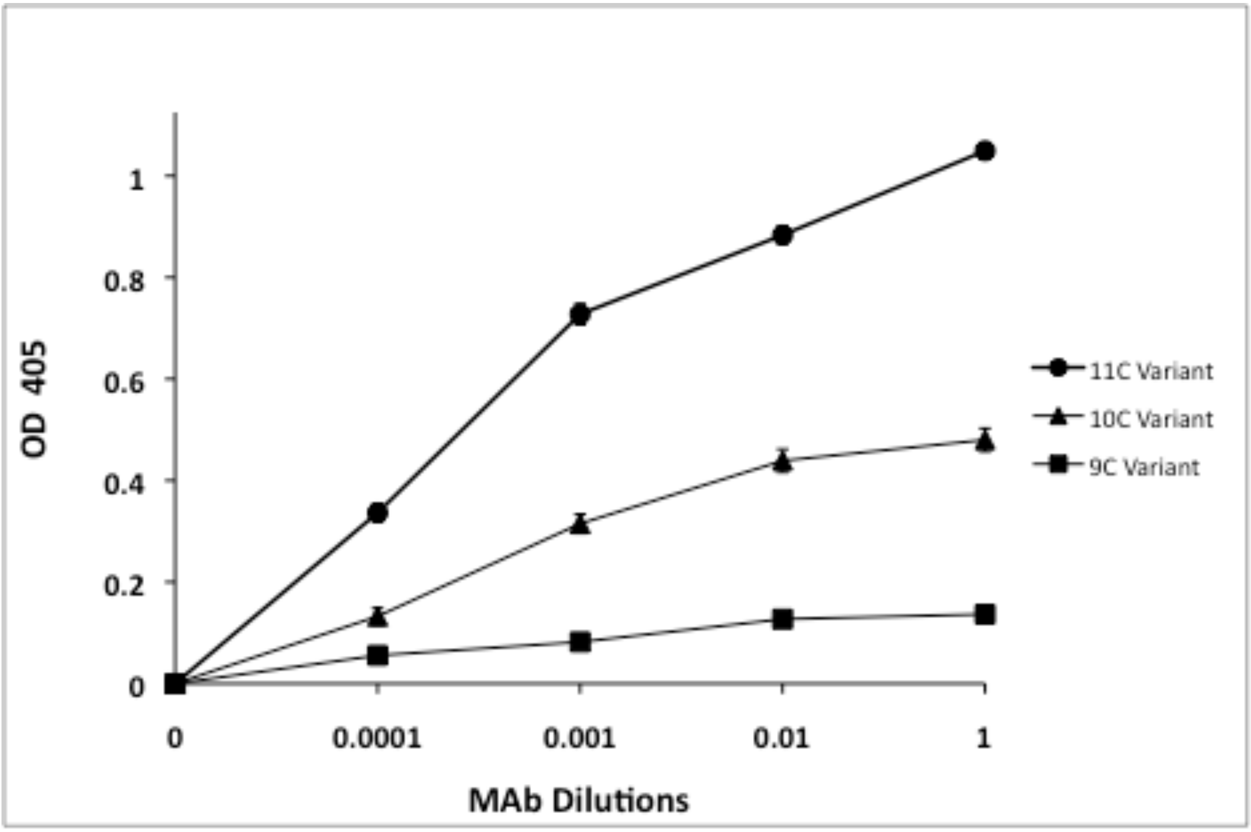

Supplementary Figure 2. Whole cell ELISA representing the level of PorA surface expression in three *porA* variants of strain 8047. Whole cells were prepared from overnight cultures of 11C, 10C and 9C *porA* variants. Cells were fixed in the presence of 0.05% Formalin, washed and resuspended in ELISA coating buffer to a final concentration of 0.5 OD550 units/ml. Cells were coated (100 μl) onto an ELISA plate and probed with a range of dilutions of mAb P1.2. Binding of mAb P1.2 was detected with an anti-mouse alkaline phosphatase conjugated antibody (1:3000 dilution) followed by substrate addition and measurement of product by taking spectrophotometer readings at OD405. Each point is the mean duplicate readings.

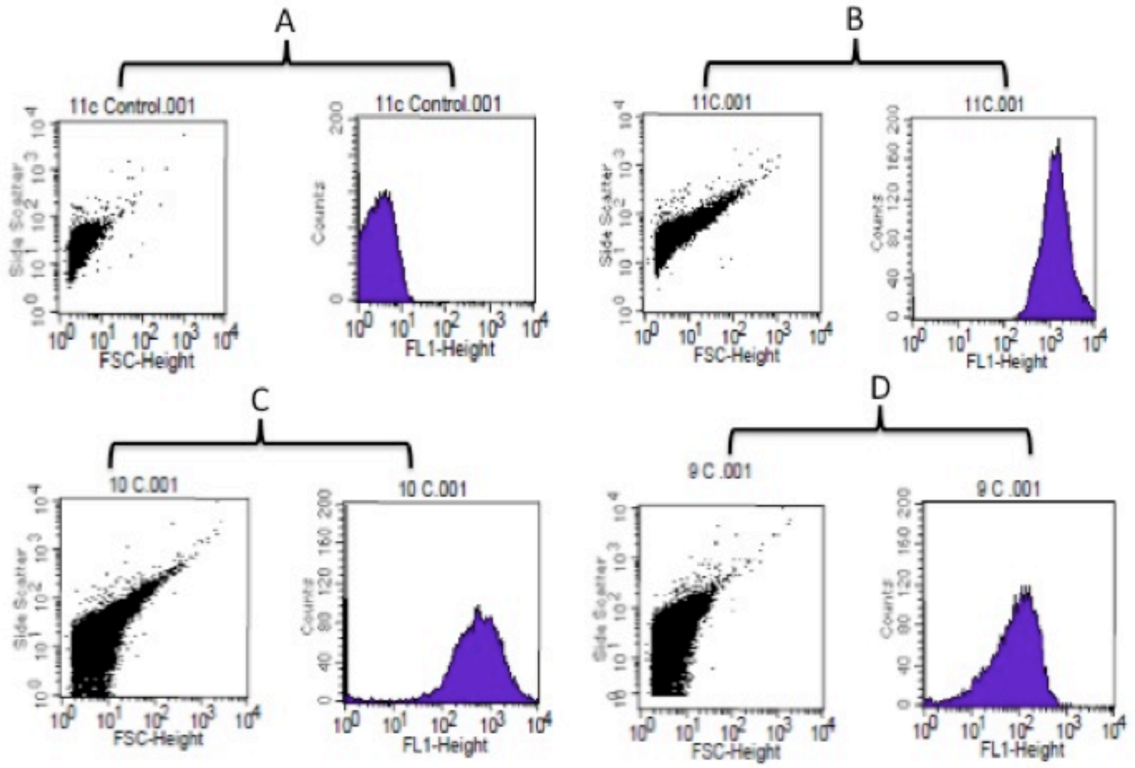

Supplementary Figure 3. Detection by FACs of binding of PorA mAb P1.2 to phase variants of *N. meningitidis* strain 8047. Meningococcal cells from an overnight culture were washed and incubated with a 1:50 dilution of mAb P1.2 in PBS/0.05%Tween-20/1% BSA followed by a 1:100 dilution of anti-mouse IgG-FITC conjugate. Meningococcal cells with bound antibody were then fixed with 0.05% formalin prior to analysis on a FACS scanner and calculation of the mean fluorescent intensity (MFI). (A) Control sample of an 11C phase variant incubated without mAb P1.2; phase variants with 11C (B), 10C (C) and 9C (D) repeat tracts incubated with mAb P1.2.

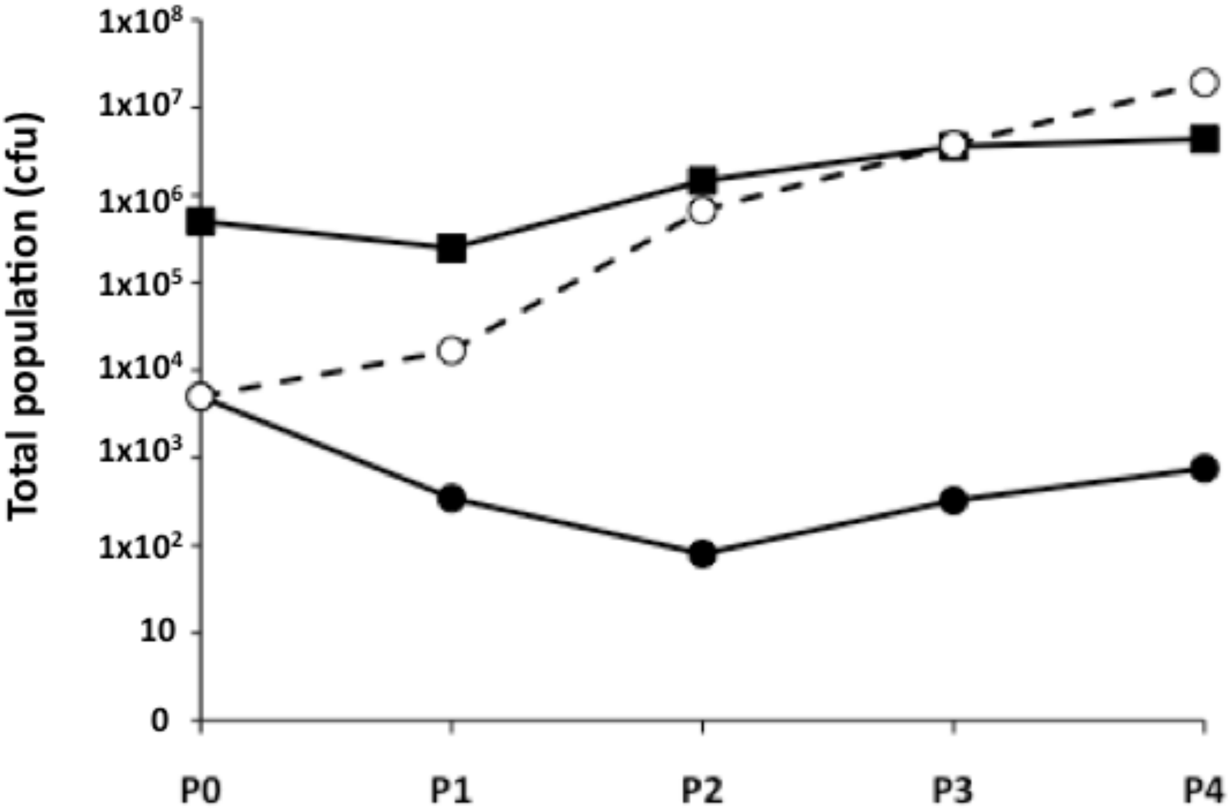

Supplementary Figure 4. **PorA expression level influences escape of PorA mAb P1.2.** Passage experiments were performed as described for figure 1 except that a 10C phase variant and 10 µl of non-diluted mAb P1.2 were utilised. Filled squares, inoculum of $5x10^5$ cfu with mAb; filled circles, inoculum of $5x10^3$ cfu, with mAb; open circles and dashed line, inoculum of $5x10^3$ cfu, no mAb.
